# Expression and Temperature-Dependent Regulation of the Beta_2_-Microglobulin (*Cyca-B2m*) Gene in a Cold-Blooded Vertebrate, the Common Carp (*Cyprinus carpio* L.)

**DOI:** 10.1155/1998/15984

**Published:** 1998

**Authors:** P. N. S. Rodrigues, B. Dixon, J. Roelofs, J. H. M. W. Rombout, E. Egberts, B. Pohajdak, R. J. M. Stet

**Affiliations:** 1 Department of Animal Sciences Cell Biology and Immunology Group Wageningen Agricultural University P.O. Box 338 Wageningen 6700 AH The Netherlands; 2 Department of Biology Dalhousie University Halifax Nova Scotia B3H 4J1 Canada

**Keywords:** Carp, MHC, beta2-microglobulin, class I, expression, temperature

## Abstract

Expression of beta_2_-microglobulin (*β*_2_m) in the common carp was studied using a polyclonal antibody raised against a recombinant protein obtained from eukaryotic expression of the *Cyca-B2m* gene. *β*_2_m is expressed on peripheral blood Ig^+^ and Ig^-^ lymphocytes, but not on erythrocytes and thrombocytes. In spleen and pronephros, dull- and bright-positive populations could be identified correlating with the presence of erythrocytes, thrombocytes, and mature leucocytes or immature and mature cells from the lympho-myeloid lineage, respectively. Thymocytes were shown to be comprised of a single bright-positive population. The Cyca-B2m polyclonal antiserum was used in conjunction with a similarly produced polyclonal antiserum to an MHC class I (*Cyca-UA*) *α* chain to investigate the expression of class I molecules on peripheral blood leucocytes (PBL) at different permissive temperatures. At 12℃, a temporary downregulation of class I molecules was demonstrated, which recovered to normal levels within 3 days. However, at 6℃, a lasting absence of class I cell-surface expression was observed, which could be restored slowly by transfer to 12C. The expression of immunoglobulin molecules on B cells was unaffected by temperature changes. The absence of the class cell-surface expression was shown to be the result of a lack of sufficient *Cyca-B2m* gene transcription, although *Cyca-UA* mRNA was present at comparable levels at all temperatures. This suggests that class I expression is regulated by a temperature-sensitive transcription of the *Cyca-B2m* gene.


*Developmental Immunology*, 1998, Vol. 5, pp. 263–275
Reprints available directly from the publisher
Photocopying permitted by license only

© 1998 OPA (Overseas Publishers Association)
N.V. Published by license under the
Harwood Academic Publishers imprint,
part of the Gordon and Breach Publishing Group
Printed in Malaysia

### Expression and Temperature-Dependent Regulation of the Beta$_2$-Microglobulin (*Cyca-B2m*) Gene in a Cold-Blooded Vertebrate, the Common Carp (*Cyprinus carpio* L.)

P. N. S. RODRIGUES[a] B. DIXON[a] J. ROELOFS[a] J. H. M. W. ROMBOUT[a] E. EGBERTS[a] B. POHAJDAK[b] and R. J. M. STET[a*]

[a]*Department of Animal Sciences, Cell Biology and Immunology Group, Wageningen Agricultural University, P.O. Box 338, 6700 AH Wageningen, The Netherlands;* [b]*Department of Biology, Dalhousie University, Halifax, Nova Scotia B3H 4J1, Canada*

*(Received 2 June 1996; In final form 14 May 1997)*

Expression of beta$_2$-microglobulin ($\beta_2$m) in the common carp was studied using a polyclonal antibody raised against a recombinant protein obtained from eukaryotic expression of the *Cyca-B2m* gene. $\beta_2$m is expressed on peripheral blood Ig$^+$ and Ig$^-$ lymphocytes, but not on erythrocytes and thrombocytes. In spleen and pronephros, dull- and bright-positive populations could be identified correlating with the presence of erythrocytes, thrombocytes, and mature leucocytes or immature and mature cells from the lympho-myeloid lineage, respectively. Thymocytes were shown to be comprised of a single bright-positive population. The Cyca-B2m polyclonal antiserum was used in conjunction with a similarly produced polyclonal antiserum to an MHC class I (*Cyca-UA*) $\alpha$ chain to investigate the expression of class I molecules on peripheral blood leucocytes (PBL) at different permissive temperatures. At 12°C, a temporary downregulation of class I molecules was demonstrated, which recovered to normal levels within 3 days. However, at 6°C, a lasting absence of class I cell-surface expression was observed, which could be restored slowly by transfer to 12°C. The expression of immunoglobulin molecules on B cells was unaffected by temperature changes. The absence of the class I cell-surface expression was shown to be the result of a lack of sufficient *Cyca-B2m* gene transcription, although *Cyca-UA* mRNA was present at comparable levels at all temperatures. This suggests that class I expression is regulated by a temperature-sensitive transcription of the *Cyca-B2m* gene.

*Keywords:* Carp, MHC, beta$_2$-microglobulin, class I, expression, temperature

### INTRODUCTION

The major histocompatibility complex (MHC) class I heterodimers are cell-surface glycoproteins comprising a heavy chain, normally referred to as $\alpha$ chain, which is associated noncovalently with $\beta_2$-microglobulin ($\beta_2$m) (Klein, 1986). In the common carp, the $\beta_2$m and class I $\alpha$ molecules are encoded by the

*Corresponding author.

*Cyca-B2m* and *Cyca-UA* genes, respectively. The Cyca-B2m mature protein has 97 amino acids and a deduced molecular weight of 11,174 Da (Dixon et al., 1993). Cyca-UA mature protein has 332 amino acids with three external domains, a transmembrane, and cytoplasmic segment (Van Erp et al., 1996a). The inferred Cyca-UA molecular weight is 37,213 Da, excluding the putative glycosylation products.

Generally, MHC class I molecules are located on the surface of most cells and are involved in binding and presentation of antigen to a subset of T lymphocytes, the cytotoxic T cells (Bjorkman et al., 1987; Salter et al., 1990). For the efficient transport of properly folded class I molecules to the cell surface, the association of the class I $\alpha$ chain with both peptide and $\beta_2$m is required (Vitiello et al., 1990). The peptide of a certain length as well as $\beta_2$m are essential not only to promote MHC class I assembly, but also to give stability to the heterodimer (Townsend et al., 1989, 1990). The free class I heavy chain that results from the dissociation of the $\beta_2$m from previously assembled heterodimers appears to be unable to present peptides to T cells (Rock et al., 1991). Although the stability of the class I heterodimer is temperature-dependent, as shown in experiments, which uses the RMA-S cell line (Ljunggren et al., 1990), nothing is known of such requirements for class I trimolecular stability in poikilothermic animals such as teleostean fish.

The immune system of fish, although with some differences, seems to be comprised of the same basic features found in other vertebrates (Turner, 1994). For some time, functional assays (e.g., skin transplantation, MLR, *in vitro* antibody production) were considered to be evidence for the presence of MHC in teleostean fish (Stet and Egberts, 1991). Recently, however, a large number of MHC (class I $\alpha$, $\beta_2$m, class II $\alpha$ and $\beta$) and MHC-related genes of different teleost species have been identified (Dixon et al., 1995)). As yet, no data are available concerning the functional properties of the molecules encoded by these MHC genes (Stet et al., 1996). Studies on function requires appropriate tools, such as antibodies. Due to availability of carp $\beta_2$m (*Cyca-B2m*) and class I $\alpha$-chain (*Cyca-UA*) full-length cDNA, it became possible to produce recombinant proteins and raise polyclonal antibodies to such proteins, as has recently been demonstrated for the Cyca-UA class I molecule (Van Erp et al., 1996a).

Experiments have shown that the immune response of fish kept at the lower limit of their physiological temperature was severely impaired (Bly and Clem, 1992). These findings were investigated further by studies carried out in the channel catfish (*Ictalurus punctatus*), where it was demonstrated that *in vitro* teleost antigen-presenting cells were able to take up, process, and present exogenous antigen and trigger antibody production at low permissive temperatures (Vallejo et al., 1992). It was concluded that the previously observed temperature-dependent suppression of primary T-cell responses in fish was not attributed to impaired class II expression. However, the effect on MHC class I molecule expression has never been investigated in fish.

In this study, the expression of class I molecules was investigated with the aid of polyclonal antibodies raised against recombinant carp class I $\alpha$-chain (Cyca-UA) and $\beta_2$m (Cyca-B2m) molecules in carp that were kept at different permissive temperatures.

### RESULTS

### Characterization of the Carp $\beta_2$-Microglobulin Antiserum

We have previously reported the cloning of a full-length cDNA encoding carp $\beta_2$-microglobulin (*Cyca-B2m*) (Dixon et al., 1993). Part (73 codons) of the *Cyca-B2m* cDNA was cloned in frame into the pRSET vector, and the resultant recombinant protein was used to immunize rabbits. The antiserum obtained was tested against the purified recombinant protein, membrane lysates of erythrocytes, and leucocytes in a western blot. The serum reacted strongly with a band of relative molecular mass ($M_r$) of 11.0 kD from the recombinant protein extraction (Figure 1), and only very weakly with a band of $M_r$ of 13.4 kD from the leucocyte membrane lysate. No reactivity was detected with membrane lysates of erythrocytes,

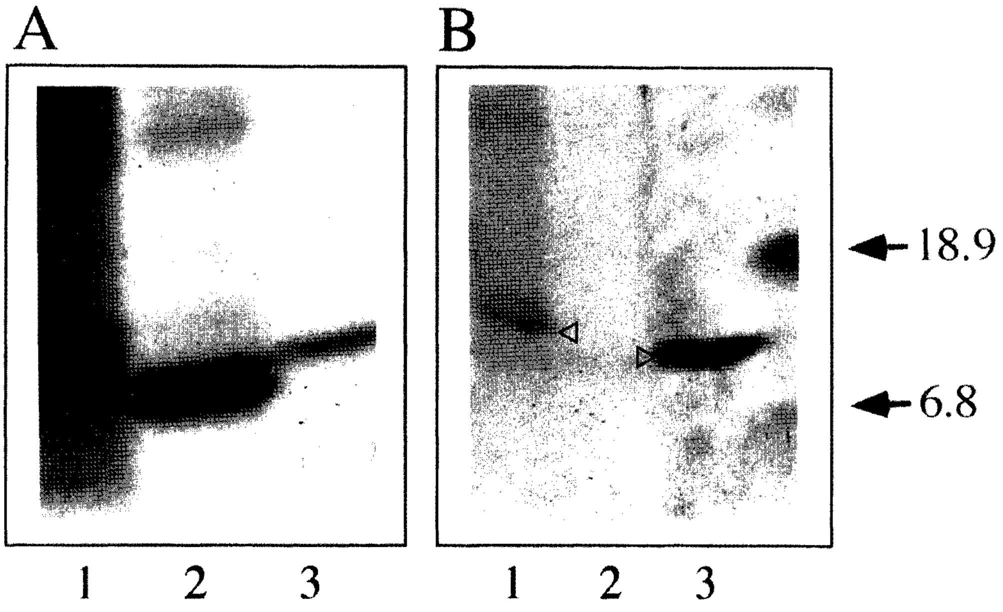

FIGURE 1 Cell membrane lysates and recombinant Cyca-B2m protein were transferred to a nitrocellulose filter and the western blot was incubated with the Cyca-B2m antiserum. (A) Coomassie stained polyacrylamide gel; PBL membrane lysate (lane 1), erythrocyte membrane lysate (lane 2), and recombinant Cyca-B2m protein (lane 3). (B) Western blot of the same samples showing reactivity of the Cyca-B2m antiserum as indicated by triangles. Arrows indicate positions of molecular weight markers in kD.

although a large amount of protein was present in the molecular weight range studied.

The antiserum was also tested on live cells by FACS analyses (Figure 2). The preimmune serum staining showed only weak reactivity, being close to the range of the conjugate only. This preimmune reactivity could be reduced by absorption to erythrocytes. The absorped antiserum was used in all subsequent experiments. The specific antiserum recognized an antigen on the cell surface of carp peripheral blood leucocytes (PBL) labeling strongly the majority (66.5%) of the leucocytes (Figure 2A). Detailed FACS analyses, using double staining of the Cyca-B2m antiserum combined with monoclonal antibodies to Ig$^+$ B cells (WCI12) and to thrombocytes (WCL6), was carried out on carp PBL (Figures 2B and 2C). The different percentages of the labeled PBL were determined from the FACS experiments. For double staining with the monoclonal against surface Ig, the percentages were Cyca-B2m$^+$/WCI12$^+$ (42%), Cyca-B2m$^+$/WCI12$^-$ (17%), Cyca-B2m$^-$/WCI12$^+$ (2%), and Cyca-B2m$^-$/WCI12$^-$ (37%). In the case of the thrombocyte marker, the following proportions were found: Cyca-B2m$^+$/WCL6$^+$ (7%), Cyca-B2m$^+$/WCL6$^-$ (57%), Cyca-B2m$^-$/WCL6$^+$ (17%), and Cyca-B2m$^-$/WCL6$^-$ (19%).

In order to study the expression of Cyca-B2m in major lymphoid organs (namely, thymus, spleen, and pronephros), leucocytes were isolated, labeled with the polyclonal anticarp $\beta_2$m, and analyzed by FACS. No expression was detected on erythrocytes, as indicated by the fact that the staining of these cells was similar to the negative control, that is, conjugate only (Figure 3). The antiserum recognized leucocytes isolated from the thymus, pronephros, and spleen, but the level of expression differed among these cell populations. Pronephrocytes and splenocytes could be divided into three populations: a negative, dull-, and bright-positive population. In the pronephros, equal representation of negative and bright-positive populations was observed, whereas with splenocytes, the majority were found in the negative and dull-positive populations. Thymocytes comprised only bright-positive cells (Figure 3B).

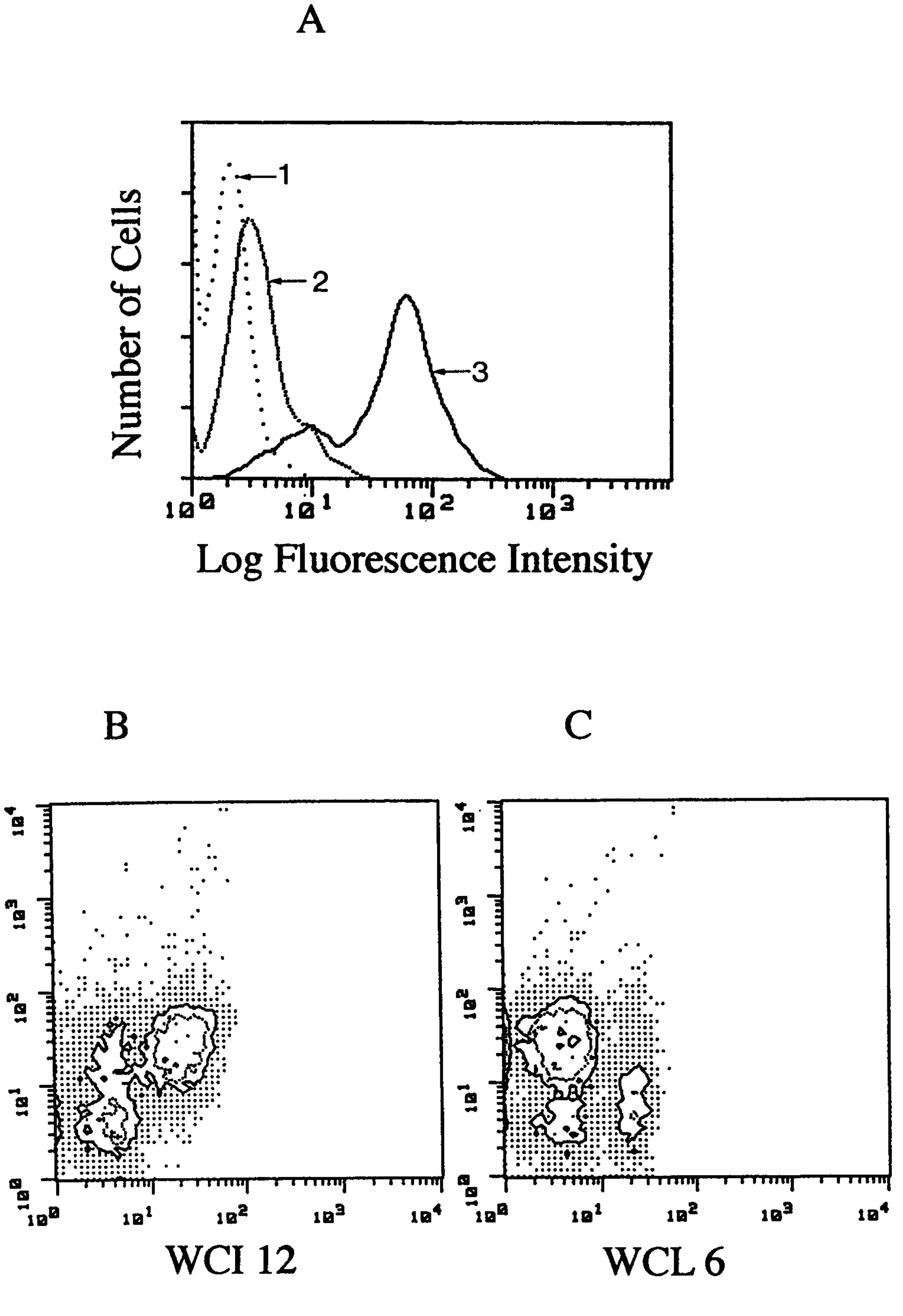

FIGURE 2 Peripheral blood leucocytes (PBL) were labeled with the anti-$\beta_2$m recombinant protein serum and the binding of the polyclonal was detected with goat anti-rabbit IgG conjugated with PE (GAM-PE). (A) The fluorescence intensity histogram depicted represents: (1) conjugate only, (2) preimmune serum, and (3) immune serum. (B and C) Fluorescence intensity contour graphs of double-labeled PBL with the Cyca-B2m antiserum, combined with the monoclonal antibody to B cells (WCI12) and to thrombocytes (WCL6). For the detection of the Cyca-B2m antiserum and the monoclonal antibodies, GAM-PE and goat anti-mouse Ig conjugated to FITC (GAM-FITC) were used, respectively.

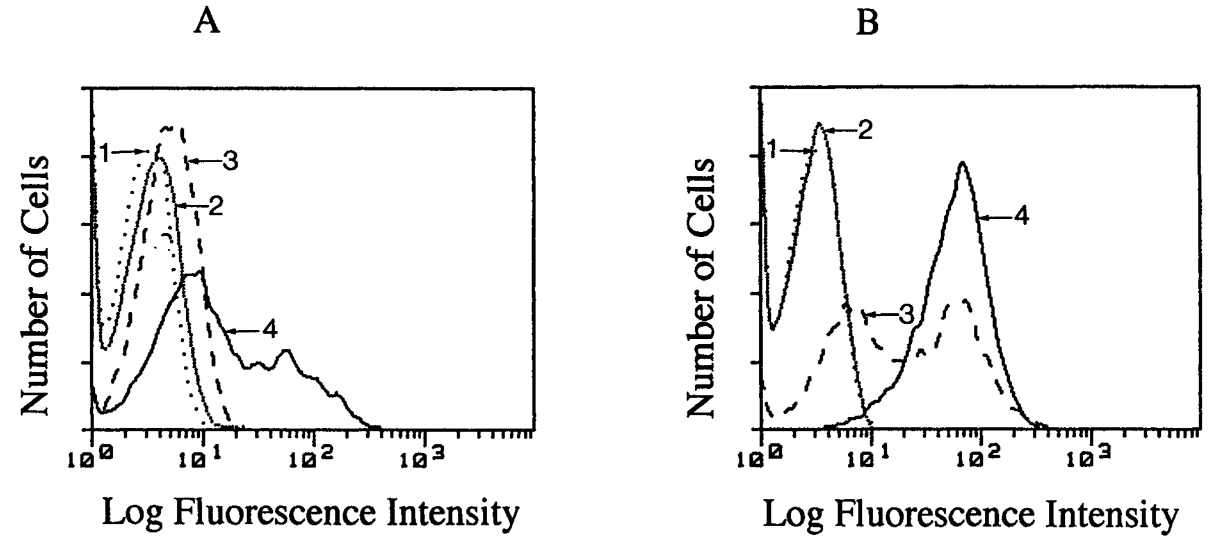

FIGURE 3 Leucocytes were isolated from cell suspensions of thymus, pronephros, and spleen. Erythrocytes were isolated from peripheral blood. Cells were labeled with the Cyca-B2m antiserum and the binding of the polyclonal was detected with GAM-PE. Fluorescence intensity histograms of cells labeled with the Cyca-B2m antiserum are shown. (A) (1) Erythrocytes control, (2) splenocytes control, (3) erythrocytes, and (4) splenocytes. (B) (1) Pronephrocytes control, (2) thymocytes control, (3) pronephrocytes, and (4) thymocytes.

### Temperature Effect on $\beta_2$-Microglobulin and MHC Class I Expression on Carp Leucocytes

MHC class I expression has been shown to be critically dependent on association with $\beta_2$m and peptide, and in RMA-S cell line, this expression can be modulated by temperature (Ljunggren et al., 1990). In this study, the effect of three different temperatures (24°C, 12°C, and 6°C) on *in vivo* cell-surface expression of MHC molecules was investigated. For this purpose, the Cyca-UA and Cyca-B2m polyclonal antiserum were used in conjunction with the WCI12 monoclonal antibody as a control (Figure 4). The antiserum raised against recombinant Cyca-UA, a locus known to be expressed, may not recognize all carp class I molecules, whereas the Cyca-B2m polyclonal antiserum is expected to give an indirect estimation of the levels of all class I cell-surface expression.

Carp PBL were isolated at different time points from the different experimental groups and labeled with the polyclonal against Cyca-B2m and Cyca-UA. In the first experiment, carp were transferred from their standard rearing temperature of 24°C to 12°C. Subsequently, they were exposed to this temperature for 2 weeks and then moved back to 24°C. Fish living at standard conditions of 24°C show the typical histograms for the three antibodies used as depicted in Figures 4 and 5 under day 0. After 3 days of exposure to 12°C, the levels of expression of Cyca-B2m and Cyca-UA were reduced, but recovered to control levels after 6 days. The expression of surface Ig during this period was constant. Transfer of carp from 12°C back to 24°C did not change all of the expression patterns studied to any significant degree (Figure 4).

In the second experiment, the carp were moved from 24°C to 12°C, and finally to 6°C. The fish remained at this temperature for 2 weeks before being transferred back to 12°C. Fish kept at 6°C showed, after 3 days at this temperature, reduced levels of Cyca-B2m and Cyca-UA expression. However, after 6 days, all cell-surface expression studied is lost and remained absent for prolonged periods, except Ig. After transferring the carp to 12°C, they recovered to normal levels within 6 days at this temperature. Cell-surface expression of surface Ig again remained constant during the experimental period Figure 5).

However, absence of Cyca-B2m and Cyca-UA at 6°C has been observed for a period up to 4 weeks.

### Temperature Effect on $\beta_2$-Microglobulin and MHC Class I Transcription in Carp Leucocytes

In the previous experiment, a number of animals were kept at the three different temperatures (24°C, 12°C and 6°C) for 4 weeks and samples for transcription analysis were taken. For each temperature group, PBL from two animals was isolated, RNA was extracted, and cDNA prepared. A PCR using specific primers for *Cyca-B2m* and *Cyca-UA* genes was carried out. The concentrations of the *Cyca-B2m* and *Cyca-UA* transcripts in PBL were compared between groups by evaluating the yield obtained at different cycle numbers (Figure 6).

Within temperature groups, no clear difference in amplification was found both for *Cyca-B2m* and *Cyca-UA*. However, between groups, a clear difference in yield of amplification was found for *Cyca-B2m*, but not for *Cyca-UA*. In the case of *Cyca-B2m* (Figure 6A), the PCR products generated from the cDNA of the 6°C temperature group were not detectable within 20 cycles, and lower yields were seen with 25 and 30 cycles when compared to the products generated in both the 24°C and 12°C temperature groups. No visible difference was found between the amount of PCR products from PBL of fish living at 24°C and 12°C. In contrast, in all temperature groups, the yield of *Cyca-UA* (Figure 6B) generated by the PCR amplification increased proportionally with the number of cycles (20, 25, and 30).

### DISCUSSION

The expression of MHC molecules and their tissue distribution cannot be dissociated from age and

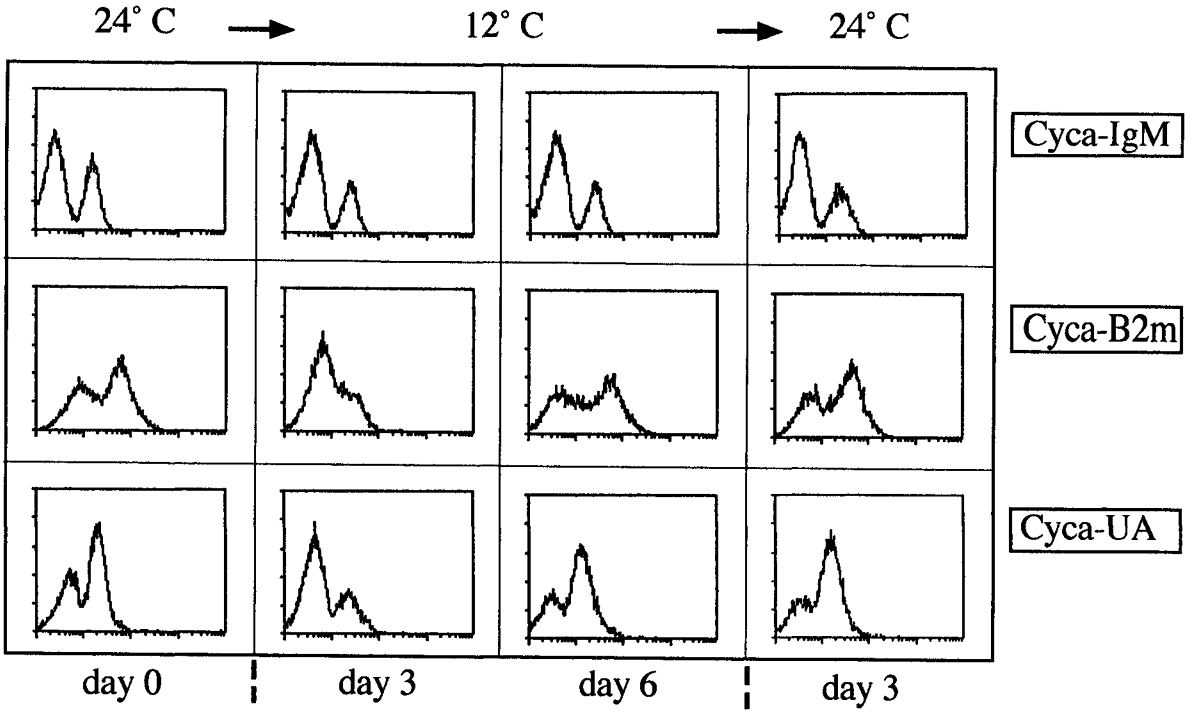

FIGURE 4 A group of carp was kept at to two different temperatures. Fish maintained at the normal temperature of 24°C were transferred to 12°C and were kept at this temperature for 2 weeks. After this period, the fish were transferred back to 24°C. Carp were bled at regular intervals, PBL were isolated and labeled with the Cyca-B2m and the Cyca-UA antisera. As a control, the WCI12, a monoclonal antibody against carp Ig was used. Labeled PBL from carp kept at different temperatures were analyzed by FACS and the relevant fluorescence intensity histograms are depicted.

immunological state of the species considered; however, it is generally accepted that class I molecules are present on the cell surface of most somatic cells, whereas class II expression occurs mainly on the cells of the immune system (Klein, 1986). With the recent isolation of carp B2m gene (*Cyca-B2m*) from a spleen/pronephros cDNA library (Dixon et al., 1993), it became possible to study the expression levels and tissue distribution of this gene. PCR amplification of cDNA obtained from leucocytes from spleen, pronephros, blood, and thymus confirmed the presence of transcripts in these organs (Rodrigues et al., in preparation). However, due to the limitations of this type of analysis, a polyclonal antiserum was produced against Cyca-B2m recombinant protein.

The Cyca-B2m antiserum showed substantial reactivity against the recombinant protein, representing only part of the native $\beta_2$m molecule. However, less reactivity could be detected with cell-surface $\beta_2$m, which as expected ran at a higher $M_r$. The weak reactivity might be explained because only approximately 50% of PBL is Cyca-B2M$^+$.

Because it is was hypothesized that Cyca-B2m is associated with all carp class I gene products, it was expected that all nucleated cells would be positive with the serum against carp $\beta_2$m. However, although nucleated erythrocytes and thrombocytes were anti-Cyca-B2m negative, and thus also class I negative. This has been confirmed by using antibodies to Cyca-UA class I molecules (Van Erp et al., 1996a). These findings are in disagreement with the distribution of *Xenopus* class I molecules, which appear at the time of metamorphosis to adult stage on leucocytes and erythrocytes (Flajnik et al., 1984, 1987). Also, in chicken, the B-F encoded molecules are expressed on nucleated erythrocytes (Møller et al., 1991). Previously, a serologically defined major histocompatibility locus K in carp has been described (Kaastrup et

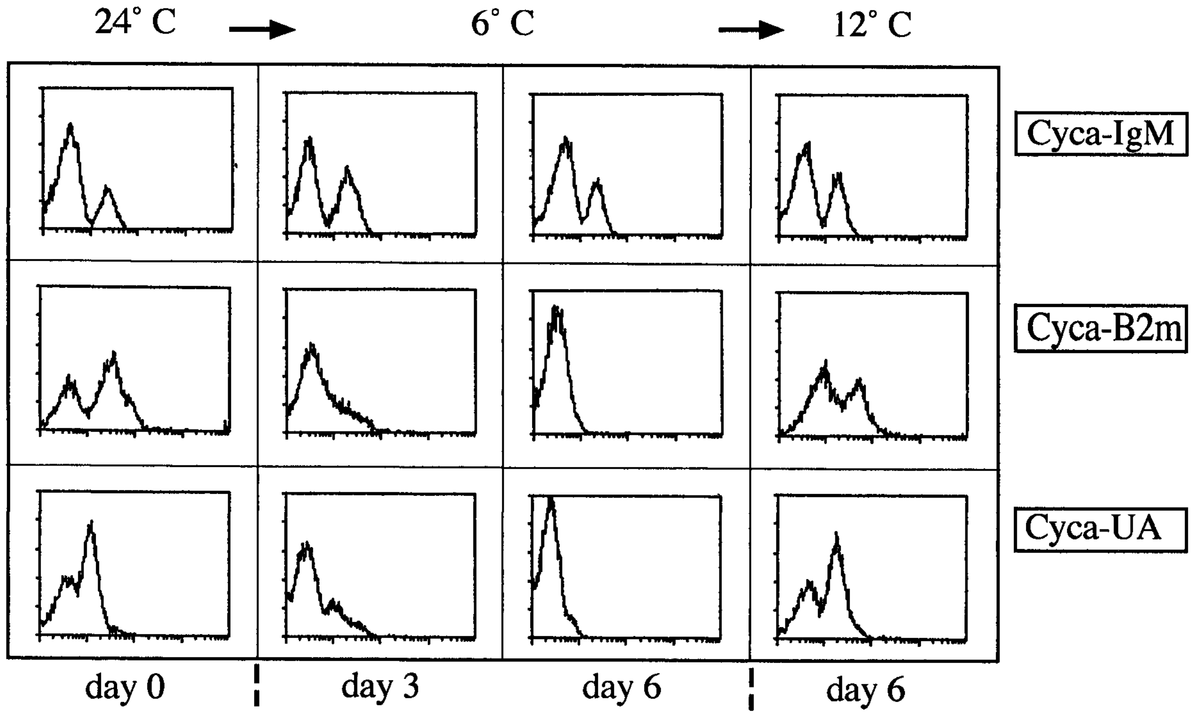

FIGURE 5 A group of carp was subjected to three different temperatures. Fish reared at the normal temperature of 24°C were transferred to 12°C and subsequently to 6°C, and were kept at this temperature for 2 weeks. After this period, the fish were transferred back to 12°C. Carp were bled at regular intervals, PBL were isolated and labeled with the Cyca-B2m and the Cyca-UA antisera. As a control, the WCI12, a monoclonal antibody against carp Ig was used. Labeled PBL from carp kept at different temperatures were analyzed by FACS and the relevant fluorescence intensity histograms are depicted.

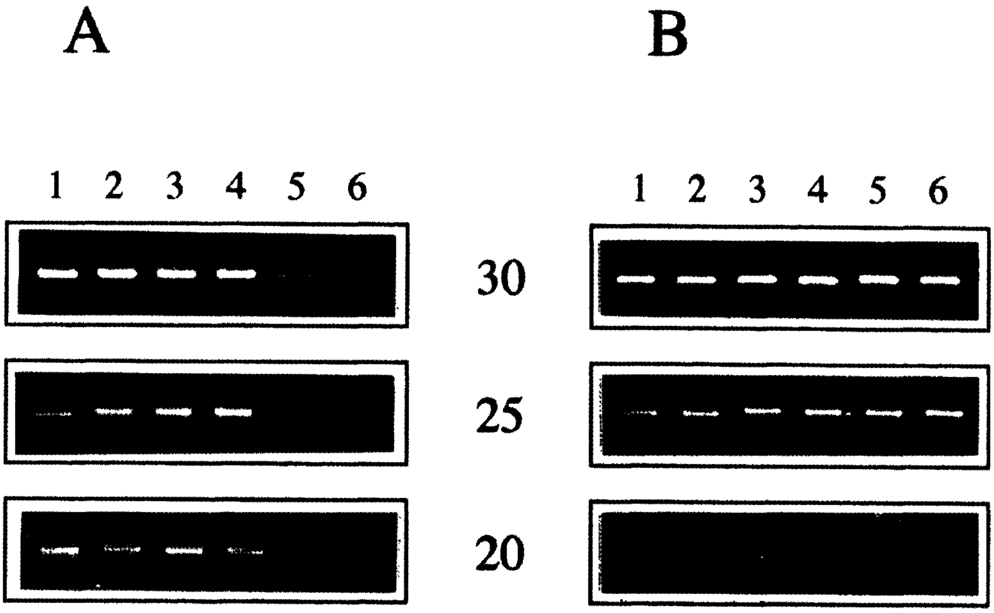

FIGURE 6 Carp were kept for 4 weeks at three different temperatures (24°C, 12°C, and 6°C). After this period, PBL were isolated from the three temperature groups. RNA was extracted, cDNA prepared, and PCR with specific primers for Cyca-B2m and the Cyca-UA was carried out and aliquots visualized by agarose gel electrophoresis. (A) PCR products for the Cyca-B2m amplification after different cycle numbers. (B) PCR products for the Cyca-UA amplification. Lanes 1 to 6 depict PCR yields from individual carp kept at different temperatures: 1 and 2 (24°C); 3 and 4 (12°C); and 5 and 6 (6°C).

al., 1989), which alleles correlated with transplant rejection, and thus were thought to ressemble class I alleles. It is clear, from the lack of $\beta_2$m expression on erythrocytes as described in this study, that the K locus is not a class I locus, but a locus that codes for an alloantigen perhaps closely linked to one of the class I major histocompatibility complex loci described in carp (Van Erp et al., 1996b).

The fluorescence histogram of labeled carp PBL showed dull- and a bright-positive populations. With the help of the only two monoclonal antibodies against carp leucocytes available, the B cell (Secombes et al., 1983) and thrombocyte markers (Rombout et al., 1996), the nature of the different populations was further investigated. The double labeling of PBL revealed that the WCL6$^+$ cells stained only weakly with the Cyca-B2m antiserum (Figure 2C). In this experiment, a population is seen that is Cyca-B2M$^-$/WCL6$^-$, which represents contaminating erythrocytes. This lack of *Cyca-B2m* expression on carp thrombocytes is in contrast to what is known of expression of class I for the mammalian counterparts of thrombocytes, the platelets (Klein, 1986) and the chicken thrombocytes (Pink et al., 1985). It was also apparent from the PBL double labeling that almost all Ig$^+$ lymphocytes (WCI12$^+$) are Cyca-B2m bright-positive. There was also an Ig$^-$ population observed that was also bright-positive, which accounts for 17% of the PBL, and are most probably putative peripheral T cells.

To investigate the tissue distribution of Cyca-B2m molecules, cells from lymphoid organs were labeled with the Cyca-B2m antiserum. Erythropoiesis and trombopoiesis occur mainly in the spleen (Rombout et al., 1996), so the proportion of thrombocytes and several stages of erythrocytes in the density-isolated cell fraction is probably high. The large proportion of dull-positive cells isolated from the spleen are most likely accounted for by the presence of thrombocytes and erythrocytes. A similar staining pattern was found with an antiserum that recognizes Cyca-UA class I molecules (Van Erp et al., 1996a). The pronephros is thought to be the hemopoietic organ in fish and therefore equivalent to the mammalian bone marrow, as well as a peripheral immunological organ containing numerous antibody-producing cells after antigenic stimulation. In such tissue, a large proportion of undifferentiated cells are present, so the dull-positive cells most likely represent the developmental stages of different leucocyte lineages, and the bright-positive cells are the mature $Ig^+$ and $Ig^-$ leucocytes. In chicken bone marrow and spleen, a similar distribution of class I (B-F) dull- and bright-positive cell populations has been reported (Dunon et al., 1990). The fluorescence histogram of the thymocytes revealed in carp just one type of cells, all of which were bright-positive. It has been demonstrated that in frogs, birds, and mammals, mainly mature thymocytes are bright-positive for class I, whereas cortical are dull-positive (Klein, 1986). A similar situation where two populations are detected in the thymus has been observed with Cyca-UA antiserum Van Erp et al., 1996a). This discrepancy may be explained because subpopulations of thymocytes use different class I $\alpha$ chains from different lineages (Van Erp et al., 1996b), which are all probably noncovalently associated with $\beta_2$m (Rodrigues et al., in preparation).

In the next set of experiments, we addressed the effect of temperature on the expression of the $\beta_2$m and Cyca-UA class I molecules. It has been demonstrated that both $\beta_2$m and peptide are instrumental in the proper folding of the trimolecular structure of class I molecules (Elliot, 1991). There are, however, instances in which either of the two requirements can be circumvented. One of the best known example is the RMA-S cell line, in which in the absence of peptides, class I expression can be rescued by lowering the temperature (Ljunggren et al., 1990). The $\beta_2$m molecule seems to be more essential for a proper class I expression (Zijlstra et al., 1990), although low levels of refolding with exogenous $\beta_2$m and peptide have been observed (Vitiello et al., 1990).

The experiments described in this study clearly showed that lowering the ambient temperature of the carp resulted in a decreased level of $\beta_2$m and class I expression, which remained undectable when carp were kept at 6°C. Recovery to normal expression levels, after transfer to a higher temperature, was achieved in all groups, albeit with a slower rate in the 6°C group. In contrast, no changes were observed in the expression of the Ig on the B cells. The latter observation is consistent with the fact that temperature effects have been noted for T-cell but not B-cell functions (Bly and Clem, 1992).

The question that arises is whether the observed downregulation of class I molecule expression is due to (1) a failure to transport the molecules to the cell surface, and (2) the inability to fold the trimolecular structure due to the absence of one or more of the constituents. The transport system of macromolecules does not seem to be impaired at low permissive temperatures because normal levels of Ig molecules have been observed on the B cells in the different temperature groups. Thus, the absence of either peptide, $\beta_2$m, or class I molecules seems to be a likely cause. Although peptide is required to fold a functional class I molecule, low temperature would allow for the expression of empty class I molecules (Ljunggren et al., 1990). In addition, the fact that antigen uptake and processing have been observed in fish at low permissive temperatures, although at a slower rate, would argue against an absence of protein-processing activities (Vallejo et al., 1992). The latter study also showed that exogenous antigens are being presented, which seems to suggest in fish a different effect of temperature on expression of class II molecules. Although peptides bound to class I or class II molecules follow different processing routes (Brodsky et al., 1996), it seems unlikely that the class I route has a different susceptibility to temperature changes compared to class II, which in fish seems to function only at a slower rate at lower temperatures.

The remaining possibility of absence of either the $\beta_2$m or the class I $\alpha$-chain molecule was investigated by semiquantative PCR. This experiment clearly indicated a downregulation of *Cyca-B2m* gene transcription only at 6°C. *Cyca-UA* gene transcription is unaffected at all temperatures. Therefore, the absence of normal transcription levels of the *Cyca-B2m* gene accounts for the downregulation of class I molecules on the cell surface. This observation is reminiscent of $\beta_2$m knockout mice, where also normal levels of class

I transcripts can be found in the absence of class I cell-surface expression (Zijlstra et al., 1990).

In conclusion, temperature changes within permissive ranges can result in temporal or longlasting changes in class I expression, which is determined by a temperature-sensitive transcription regulation mechanism of the $\beta_2$m gene. This observation could account, in part in aquacultural practices, for the observed "immunological disasters" after severe and sudden temperature changes (Bly and Clem, 1992). This applies especially to those cases where CTL responses are required for a proper immunological response to the causative agent, such as might be the case in "winter saprolegniosis" (Bly et al., 1992) or perhaps more importantly in viral infections.

### MATERIALS AND METHODS

### Animals

Common carp (*Cyprinus carpio* L.) were reared at 23°C in recirculating UV-sterilized water, and fed pelleted dry food (Provimi, Rotterdam, The Netherlands) at a ratio of 2% of body weight per day. Animals from a single F1 hybrid family R3 × R8, weighing between 150-250 g (10-16 months), were used. R3 and R8 are partly inbred strains of common carp originating from Poland and Hungary, respectively (Wiegertjes et al., 1994).

### Production of Recombinant Protein and Polyclonal Antisera

A PCR fragment was produced by anchored PCR using the previously described *Cyca-B2m* cDNA clone in conjunction with the specific primer OL-85 (Dixon et al., 1993). This fragment, which contained the last 73 codons and the 3′ untranslated region of the clone, was cloned into the vector pCRII and sequenced using the Sequenase version 2.0 kit (U.S. Biochemical). Following sequence confirmation, this fragment was excised from the vector using *Eco*RI and ligated in frame into the vector pRSET (Invitrogen, Leek, The Netherlands). The plasmid was transformed into bacterial strain BL21-DE3 (Novagen, Madison, WI) and recombinant protein production was induced using 0.8 mM IPTG. The recombinant B2m protein was purified by nickel-affinity chromatography using the Xpress system (Invitrogen). The recombinant B2m protein was further purified over Sephadex G-100 to remove contaminating bacterial proteins. Under denaturating conditions, SDS-PAGE was used to determine the purity and size of the recombinant protein. Four hundred micrograms of the protein were conjugated to 2 mg of KLH by glutaraldehyde and were injected i.m. into a rabbit at two sites in combination with complete Freund's adjuvant. The animal was boosted with antigen and incomplete Freund's adjuvant after 1 month and bled at 2 months postinjection. Serum was collected after clotting and stored at −80°C for future use.

### Western Blotting

Membrane lysates were prepared from both peripheral blood leucocytes and erythrocytes. Briefly, cells ($10^8$) were subjected to three consecutive freeze-thaw cycles in PBS, adjusted to 270 mOsm, containing 1 mM $MgCl_2$. After each cycle, membrane fragments and nuclei were collected by centifugation at 13,000 g. After the last cycle, membranes were solubilized in lysisbuffer (10 mM Tris/HCl, pH 8, 1 mM $MgCl_2$, 150 mM NaCl, 0.1 mM protease inhibitors, and 1% CHAPS). Nuclei were removed by centrifugation at 13,000 g and lysates stored at −80°C for futher use. The recombinant protein was isolated on a preperative SDS polyacrylamide gel (Prep-gel, Biorad). The protein samples, obtained after this producere, were separated on a 10-20% SDS polyacrylamide gel. The protein was transferred to a nitrocellulose filter by electroblotting and the filter was incubated with the polyclonal antisera at an appropriate dilution. Reactivity of the rabbit serum was determined using an alkaline phosphatase conjugated goat anti-rabbit immunoglobulin and visualized using and NBT and BCIP substrate buffer.

### Cell Isolation

Fish were anesthetized in tricaine methane sulphonate (TMS, Crescent Research Chemicals, Phoenix, AZ) at

3 g/10 l, and heparinized blood was collected from the dorsal aorta. This was diluted 1:1 in cRPMI (RPMI 1640 adjusted to 270 mOsm), and peripheral blood leucocytes were separated on Lymphoprep (Nycomed, Oslo, Norway) by centrifugation at 840 g for 30 min at 4°C. The cells were harvested from the interface, washed twice, and resuspended in cRPMI. The peripheral blood leucocytes were used for further analyses. Pronephros, spleen, and thymus-cell suspensions were prepared by forcing the tissues through a 50-mesh nylon gauze filter while adding cRPMI. After being washed in cRPMI (680 g for 10 min at 4°C) and resuspended, the cell suspensions were separated on Lymphoprep by centrifugation at 840 g for 30 min at 4°C. The cells were harvested, washed twice, and resuspended at a concentration of $10^7$ cells/ml in cRPMI.

### Flow Cytometry (FACS)

For the flowcytometry studies, the following antibodies were used: the polyclonal described in this paper raised against recombinant Cyca-B2m protein; an anti-class I (Cyca-UA) polyclonal serum (Van Erp et al., 1996a); WCI12, a monoclonal antibody that detects carp surface immunoglobulin (Secombes et al., 1983); WCL6, a monoclonal antibody specifically recognizing carp thrombocytes (Rombout et al., 1996). Peripheral blood leucocytes (PBL), isolated as described earlier, were incubated for 30 min on ice in 0.5 ml of appropriately diluted (usually 1:100) Cyca-UA or Cyca-B2m polyclonal antibodies. For all the incubation and washing steps, FACS medium containing cRPMI, 1% BSA, and 0.1% $NaN_3$ was used. After washing, binding of the polyclonal antibodies was detected by incubating the cells for 15 min on ice with phycoerythrine (PE) conjugated goat anti-rabbit immunoglobulin antibody (GAR-PE; Southern, Birmingham, AL), diluted 1:100 in FACS medium, containing 1% of pooled carp serum. For the PBL double labeling, leucocytes were incubated with the Cyca-B2m polyclonal antiserum together with WCI12 or WCL6, followed by an incubation with GAR-PE and fluorescein isothiocyanate (FITC) conjugated goat anti-mouse immunoglobulin serum (Dakopatts, Denmark). Cells were analyzed using a FACStar (Becton Dickinson Immunocytometry Systems, Mountain View, CA) with an argon laser tuned at 488 nm. For analyzing the FACS results, the Consort 30 data analysis package was used.

### Temperature Experiments

A group of eighteen carp from the same F1 generation (R3 × R8) were kept at three different temperatures. The control group of six individuals was maintained at normal rearing conditions, that is, at a temperature of 24°C throughout the experiment. The remaining twelve individuals were kept at 12°C. After 3 days, six animals from the 12°C group were transferred to 6°C. The resulting three groups of six animals were kept at these temperatures for at least 2 weeks. After this period, the carp at 12°C were transferred to 24°C, and the animals at 6°C were placed at 12°C. Experimental fish were bled at regular intervals, and isolated PBL were labeled with different sera and analyzed by FACS. Small numbers of carp were kept at the three different temperatures for up to 1 month, after which they were sacrificed for further transcription analyses using RNA isolation procedures.

### RNA and cDNA Preparation

RNA and cDNA preparation for the use in semi-quantitative PCR analyses have been performed as described by Rodrigues et al. (1995). Briefly, cells were thawed out in lysis buffer (4 M guanidium thiocyanide, 25 mM sodium citrate, pH 7.0, 0.5% sarcosyl, 0.1 M 2$\beta$-ME) followed by phenol/chloroform extractions. Total RNA was precipitated in ethanol, washed and dissolved in water, and stored at −80°C. Samples containing 10 mg of total RNA were converted into cDNA using the Riboclone cDNA Synthesis System (Promega, Madison, WI). Efficiency of cDNA synthesis was traced by determining the incorporation of fluorescein-dUTP in a parallel reaction. The labeled cDNA samples were serially diluted and blotted onto a nylon filter (Hybond N+, Amersham, UK). Detection was carried out by an enzyme-linked immunoassay using an anti-fluorescein alkaline phosphatase conjugate, subsequent addition of a chemiluminescent detectionreagent (Amersham), and exposure to XAR5 film (Kodak) for 16 hr at room temperature.

### Polymerase Chain Reaction (PCR)

In order to detect *Cyca-B2m*, transcripts by PCR, two oligonucleotides 5′-ATG AGA GCA ATC ATC ACT TTT GC-3′ starting at codon 1, and 5′-TTA CAT GTT GGG CTC CCA AA-3′ ending at codon 98 were produced based on *Cyca-B2m* sequences (2). Similarly, for the detection of *Cyca-UA* transcripts, two oligonucleotides 5′-GGT GTT CAC TCA GTC CAG-3′ starting at codon 1 of $\alpha_2$ domain, and 5′-GCG CCT GCA GTT TTG ATC TTG TCC-3′ ending at codon 96 of the $\alpha_3$ domain were produced based on *Cyca-UA* cDNA sequences (Van Erp et al., 1996a). The amplification was performed in *Taq* buffer (Eurogentec, Seraing, Belgium), using 1 unit of Goldstar *Taq* polymerase (Eurogentec), supplemented with 1.5 mM $MgCl_2$, 0.2 mM of each primer, and 200 mM of each dNTP in a final volume of 100 ml. Template concentrations were balanced according to the levels of Fl-dUTP incorporated into cDNA in a parallel reaction (see "RNA and cDNA Preparation"). The mixtures were subjected to a thermal cycle profile (1 min, 94°C; 2 min, 55°C; and 1 min, 72°C) for a different number of cycles and analyzed by agarose gel electrophoresis.

### *Acknowledgements*

This work was supported by grants from the Junta Nacional de Investigação Cientifica e Tecnológica (P.N.S. Rodrigues-BD/1705/91-ID) and the Natural Sciences and Engineering Research Council (B. Pohajdak and Brian Dixon). The authors would like to thank Jim Kaufman for technical advice, and Laura M'Rabet and Trudi Hermsen for their assistance in part of the experiments.

### *References*

Bjorkman P.J., Saper M.A., Samraoui B., Bennett W.S., Strominger J.L., and Wiley D.C. (1987). The foreign antigen binding site and T cell recognition regions of class I histocompatibilty antigens. Nature **329**: 512-518.

Bly J.E., and Clem L.W. (1992). Temperature and teleost immune functions. Fish and Shellfish Immunol. **2**: 159-171.

Bly J.E., Lawson L.A., Dale D.J., Szalai A.J., Durborow R.M., and Clem L.W. (1992). Winter saprolegniosis in channel catfish. Dis. Aquat. Org. **13**: 155-164.

Brodsky F.M., Lem L., and Bresnahan P.A. (1996). Antigen processing and presentation. Tissue Antigens **47**: 464-471.

Dixon B., Stet R.J.M., van Erp S.H.M., and Pohajdak B. (1993). Characterization of $\beta_2$-microglobulin transcripts from two teleost species. Immunogenetics **38**: 27-34.

Dixon B., van Erp S.H.M., Rodrigues P.N.S., Egbert E., and Stet R.J.M. (1995). Fish major histocompatibility complex genes: An expansion. Dev. Comp. Immunol. **19**: 109-135.

Dunon D., Salomonsen J., Skjødt K., Kaufman J., and Imhof B.A. (1990). Ontogenic appearance of MHC class I (B-F) antigens during chicken embryogenesis. Dev. Immunol. **1**: 127-135.

Élliott T. (1991). How do peptides associate with MHC class I molecules. Immunol. Today **12**: 386-388.

Flajnik M.F., Hsu E., Kaufman J.F., and Du Pasquier L. (1987). Changes in the immune system during metarmorphosis of Xenopus. Immunol. Today **8**: 58-64.

Flajnik M.F., Kaufman J.F., Riegert P., and Du Pasquier L. (1984). Identification of class I major histocompatibility complex encoded in the amphibian *Xenopus*. Immunogenetics **20**: 433-442.

Kaastrup P., Stet R.J.M., Tigchelaar A.J., Egberts E., and Van Muiswinkel W.B. (1989). A major histocompatibility locus in fish: Serological identification and segregration of transplantation antigens in the common carp (*Cyprinus carpio* L.). Immunogenetics **30**: 284-290

Klein J. (1986). The Natural History of the Major Histocompatibility Complex (New York: John Wiley).

Ljunggren H.G., Stam N.J., Ohlen C., Neefjes J.J., Hoglund P., Heemels M.T., Bastin J., Schumacher T.N.M., Townsend A., Karre K., and Ploegh H.L. (1990). Empty MHC class I molecules come out in the cold. Nature **346**: 476-480.

Møller L.B., Kaufman J., Salomonsen J., Avila D., Lambris J.D., and Skjødt K. (1991). Variations in the cytoplasmic region account for the heterogeneity of the chicken MHC class I (B-F) molecules. Immunogenetics, **34**: 110-120.

Pink J.R.L., Kieran M.W., Rijnbeek A.M., and Longenecker B.M. (1985). A monoclonal antibody against chicken MHC Class I (B-F) antigens. Immunogenetics **21**: 293-297.

Rock K.L., Gamble S., Rothstein L., Gramm C., and Benacerraf B. (1991). Dissociation of $\beta_2$-microglobulin leads to a substantial pool of inactive class I MHC heavy chains on the cell surface. Cell **65**: 611-620.

Rodrigues P.N.S., Hermsen G.T., Rombout J.H.W.M., Egbert E., and Stet R.J.M. (1995). Detection of MHC Class II transcripts in lymphoid tissues of the common carp (*Cyprinus carpio* L.). Dev. Comp. Immunol. **19**: 483-496.

Rombout J.H.W.M., Koumans-van Diepen J.C.E., Emmer P.M., Taverne-Thiele J.J., and Taverne N. (1996). Characterization of carp thrombocytes with specific monoclonal antibodies. J. Fish Biology **49**: 521-531.

Salter R.D., Benjamin R.J., Wesley P.K., Buxton S.E., Garrett T.P.J., Clayberger C., Krensky A.M., Norment A.M., Littman

D.R., and Parham P. (1990). A binding site for the T-cell co-receptor CD8 on the $a_3$ domain of HLA-A2 histocompatibility antigens. Nature **345**: 41-46.

Secombes C.J., van Groningen J.J.M., and Egberts E. (1983). Separation of lymphocyte subpopulations in carp by monoclonal antibodies: immunohistochemical studies. Immunology **48**: 165-175.

Stet R.J.M., Dixon B., van Erp S.H.M., van Lierop M.C., Rodrigues P.N.S., and Egberts E. (1996). Inference of structure and function of fish Major Histocompatibility Complex (MHC) molecules from expressed genes. Fish and Shellfish Immunol. **6**: 305-318.

Stet R.J.M., and Egberts E. (1991). The histocompatibility system in teleostean fishes: From multiple histocompatibility loci to a major histocompatibility complex. Fish and Shellfish Immunol. **1**: 1-16.

Townsend A., Elliott T., Cerundolo V., Foster L., Barber B., and Tse A. (1990). Assembly of MHC class I molecules analysed in vitro. Cell **62**: 285-295.

Townsend A., Ohlen C., Bastin J., Ljunggren H.-G., Foster L., and Karre K. (1989). Association of class I major histocompatibility heavy and light chains induced by viral peptides. Nature **340**: 443-448.

Turner R.J. (1994). Immunology: A Comparative Approach (New York: John Wiley).

Vallejo A.N., Miller N.W., and Clem L.W. (1992). Cellular pathway(s) of antigen processing in fish APC: Effect of varying in vitro temperatures on antigen catabolism. Dev. Comp. Immunol. **16**: 367-381.

Van Erp S.H.M., Dixon B., Figueroa F., Egberts E., and Stet R.J.M. (1996a). Identification and characterisation of a new major histocompatibility complex class I gene from carp (*Cyprinus carpio* L.). Immunogenetics **44**: 49-61.

Van Erp S.H.M., Egberts E., and Stet R.J.M. (1996b). Evidence for multiple distinct major histocompatibility complex class I lineages in teleostean fish. Eur. J. Immunogenetics **23**: 371-381.

Vitiello A., Potter T.A., and Sherman L.A. (1990). The role of $\beta_2$-microglobulin in peptide binding by class I molecules. Science **250**: 1423-1426.

Wiegertjes G.F., Stet R.J.M., and van Muiswinkel W.B. (1994). Divergent selection for antibody production in common carp (*Cyprinus carpio* L.) using gynogenesis. Animal Genetics **25**: 251-257.

Zijlstra M., Bix M., Simister N.E., Loring J.M., Raulet D.H., and Jaenisch R. (1990). $\beta_2$-microglobulin deficient mice lack $CD4^-8^+$ cytolytic T cells. Nature **344**: 742-746.

